# BASIC PSYCHOLOGICAL NEED SATISFACTION AND WELL-BEING IN GRANDPARENT–ADULT GRANDCHILD DYADS

**DOI:** 10.1093/geroni/igae098.0607

**Published:** 2024-12-31

**Authors:** Yue Yang Sun, Xiaoye Liu, Yukai Zhang, Tianyuan Li

**Affiliations:** The Chinese University of Hong Kong (Shenzhen), Shenzhen, Guangdong, China (People’s Republic); The Chinese University of Hong Kong (Shenzhen), Shenzhen, Guangdong, China (People’s Republic); The Chinese University of Hong Kong (Shenzhen), Shenzhen, Guangdong, China (People’s Republic); The Chinese University of Hong Kong (Shenzhen), Shenzhen, Guangdong, China (People’s Republic)

## Abstract

Past research reveals that basic psychological need satisfaction (BPNS) in relationship is an important contributor to relationship intimacy and individuals’ psychological well-being, but few studies explored the effects in grandparent-adult grandchildren relationship. The current study recruited 294 adult grandchild-closest grandparent dyads from mainland China. The grandchild and the grandparent completed a survey, respectively. We adopted Actor-Partner Interdependence Model to estimate both the actor and partner effects of BPNS on each partner’s relationship intimacy and psychological well-being while controlling relevant demographic variables. Results indicated that both grandparents’ and grandchildren’s BPNS in the relationship was related to their own higher relationship intimacy, bs =.20 and.27, ps <.001, higher life satisfaction, bs =.32 and.17, ps <.03, and fewer depressive symptoms, bs = -.17 and -.12, ps <.001. Further, grandparents’ BPNS was also related to their grandchildren’s higher life satisfaction, b =.21, p =.01, and less depressive symptoms, b = -.07, p =.04, but adult grandchildren’s BPNS was not related to grandparents’ psychological well-being, ps >.13. The findings indicate the significant role that BPNS plays in promoting relationship intimacy in the grandparent-adult grandchild relationship as well as in contributing to both partners’ psychological well-being. More importantly, the support from adult grandchildren to grandparents not only benefits the grandparent’s psychological well-being, but also the grandchild’s. The asymmetric effects suggest that the grandchildren may become the more influential support provider in their relationship with the grandparent when they have grown up.

